# Editorial: Reimagining animal sheltering: Support services and community-driven sheltering methods

**DOI:** 10.3389/fvets.2022.1011202

**Published:** 2022-09-13

**Authors:** Peter Joseph Wolf, Julie Levy, E. Susan Amirian, Kevin Horecka

**Affiliations:** ^1^Enterprise Data and Analytics, Best Friends Animal Society, Kanab, UT, United States; ^2^Maddie's Shelter Medicine Program, College of Veterinary Medicine, University of Florida, Gainesville, FL, United States; ^3^Department of Research, Austin Pets Alive!, Austin, TX, United States

**Keywords:** One Health, companion animals, animal welfare, animal sheltering, community services, sustainable development

In the original call for submissions to this Research Topic, we highlighted the relative lack of research into various aspects of community-based animal sheltering, a set of sheltering principles and methods grounded in the belief that domesticated (and especially companion) animals are part of a larger system of people, animals, and the environment (often referred to as One Health) whose sustainability, stability, and health are dependent upon more direct participation of the community in animal-related services. In effect, community-based animal sheltering seeks to disperse programs and practices that would normally be housed in, and restricted to, a brick-and-mortar shelter facility throughout the geography and demography of a city, and to understand the connections between the socio-cultural structures of a society and its animal residents.

Because such a holistic approach invites—perhaps necessitates—innovative methods and novel measures of success, we anticipated that community-based animal sheltering would be a topic of considerable interest to researchers and animal sheltering practitioners. We were not disappointed, therefore, to see no fewer than 20 articles published in this volume.

Each article illuminates a particular aspect of community-based animal sheltering, a broad topic and one that is new enough to resist any easy definition. Some focus their attention squarely at the intersection of “traditional” animal services and community engagement (e.g., emergency fostering of dogs during the COVID-19 pandemic), while the focus of others is mostly **one** or the other. Similarly, some articles report on conditions “as they are” in shelters and the communities they serve (e.g., free-roaming dog populations), while others report on operational or programming outcomes (e.g., an increased live release rate as the result of community cat programming). The diversity of research questions addressed, and methods employed, is a testimony to the complexity of this Research Topic.

Horecka and Neal's conceptual analysis sets the stage, providing a big-picture view of “critical problems for research in animal sheltering” broken down into seven key areas, each with its own potential impacts. The authors' analysis combines input from more than 300 animal sheltering professionals and an extensive review of the relevant literature. They note that many of the key areas they have identified “are being actively worked upon by various research institutions (i.e., significant work in animal diseases has occurred), but some have received little attention yet (i.e., operations research).” Their fellow contributors to this Research Topic have helped fill in at least some such gaps.

## Shelter operations and programs

Using a “qualitative, comparative ethnographic study that included semi-structured interviews, participant observation, and archival research,” Thomsen et al. examined the potential role of social entrepreneurship in improving shelter outcomes. Their findings describe some of the ways animal shelters are adopting more business-like practices (e.g., professionalizing shelter management, creating a welcoming retail experience for visitors), resulting in changes that can benefit staff, volunteers, and—ultimately—the animals in their care.

Hurley discusses the trend toward “triage and appointment-based services” in animal shelters and their positive results. Whereas triage “is a well-developed strategy in human general practice medicine,” animal shelters have typically permitted the “unscheduled admission of any animal presented during open hours… regardless of shelter capacity or animal needs.” However, recent changes in admission policies and practices, prompted in many cases by restrictions related to the COVID-19 pandemic, have revealed numerous benefits (e.g., reduced euthanasia, more predictable workflow, reduced disease transmission). *Ad hoc* shelter admissions of cats, in particular, has often led to euthanasia. “In North America alone,” explain Hurley and Levy, “hundreds of millions of cats have been impounded and euthanized and billions of dollars invested in such programs.” The authors compare this “traditional” method with **two** alternatives: a shelter-based trap-neuter-return (TNR) program, and “leaving cats in place with or without referral to mitigation strategies or services provided by other agencies.”

Among the many shelters to implement appointment-based admissions and shelter-based TNR is Memphis Animal Services, in Memphis, Tennessee. Their adoption was part of a larger shift toward improved lifesaving that began in 2017. Kreisler et al. examined the results of this shift, reporting, for example, that the shelter's live release rate for cats increased from 62% in 2016 to a median of 92% post-intervention. Improvements for canine live release rate were more modest, from roughly 75% in 2016 to “just below 90% for 2017 through 2021.” Post-intervention, euthanasia numbers were no longer closely correlated with admission numbers for either species.

## Community-based programs and services

Measuring the effectiveness of community-based programs and services is critical to their success. Hawes et al. employed six questions from the One Health Community Assessment to “measure perceptions of access to pet care in two urban and two rural zip codes.” Residents of one urban and one rural zip code received community-based animal welfare services (e.g., low- or no-cost veterinary services, pet food delivery, collars and leashes), while residents of the other zip codes did not. In the urban communities, residents who received community-based services reported “a higher overall measure of access to pet care” than their urban counterparts who received no such services. This was not true among the rural residents, however.

Using 2013–2020 pet food bank records from the British Columbia Society for the Prevention of Cruelty to Animals, Schor and Protopopova examined temporal trends, paying particular attention to any potential impacts of the COVID-19 pandemic. Among their findings, some were anticipated (e.g., the number of clients receiving services in 2020 was significantly less than in previous years), while others were not (e.g., cat owners received the largest share of services).

## Programs and services at the intersection of shelter and community

As more shelters come to recognize the potential impact of community-based programs and services, they are beginning to let go of more “traditional” sheltering practices. During the early months of the COVID-19 pandemic, media accounts reported on the surge in foster caregiving (1–3). Gunter et al. examined the phenomenon at 19 US shelters, finding that foster caregiving increased during the first 2 months but settled back to initial levels by June 2020. Nearly 40% of caregivers had no prior experience fostering dogs for their local shelter. Shelters with fewer resources tended to rely on known foster caregivers and transfer dogs to other agencies, whereas more highly resourced shelters tended to recruit new fosters and place dogs with adopters in their communities.

Kremer developed a web-based tool designed to improve canine return-to-owner (RTO) rates, using geographical data from Dallas (Texas) Animal Services to demonstrate its usefulness. The subsequent analysis showed that 70% of stray dogs reunited with their owners were at most 1 mile from their home, while 42% were within a block of their home. The RTO rate for adult dogs with microchips was 71%, compared to 39% for those without microchips.

Using the Canadian Index of Multiple Deprivation (CIMD), Ly et al. “compared the ‘flow' of surrendered animals between originating communities (incoming) and communities where they were adopted (outgoing).” Their results reveal a flow that is often unbalanced, with animals moving from more vulnerable to less vulnerable communities. The authors' findings “provide a basis for understanding potential inequities in the use of shelter services to surrender or adopt an animal” and the development of interventions that can better balance the flow between communities.

## Shelter conditions

Rodriguez et al. examined intake and outcome data from 1,373 US animal shelters over a five-year period (2016–2020). Their analysis shows that intake and euthanasia significantly decreased over this period, for both dogs and cats. Meanwhile, live release rates increased significantly for both species. A number of live outcome categories—adoptions, return-to-owner, return-to-field, and transfers to other agencies (for cats), each as a proportion of total intake—showed significant increases as well.

Although 51.1% of US shelter admissions during 2020 were dogs, cats made up 68.4% of the animals “unnecessarily dying” there (4). Using structural equation modeling, Kilgour and Flockhart predicted that cat outcomes at a Washington, DC, animal shelter could be predicted on the basis of four interrelated factors: characteristics of the cats (e.g., sex, coat pattern and color, health status); where the cat came from, the date and type of intake (owner-surrendered, stray), and the cat's length of stay in the shelter. “Consistent with other studies,” the authors report that, “intake type, potentially indicating degree of ownership, and physical attributes of cats are both important components of the system relating to outcomes.”

One topic of increasing interest in recent years has been the difficulties associated with shelters recruiting and retaining veterinary professionals (5, 6). Powell et al. surveyed 52 shelter veterinarians, along with 39 former shelter veterinarians and 130 veterinarians working in private practice, in their investigation of the “characteristics of employment in shelter medicine relative to turnover or retention of shelter veterinarians.” The authors report that veterinarians who “participate in decision-making for patients and shelter management procedures” are more likely to be retained by shelters than their colleagues who aren't offered such opportunities.

## Community context

To better understand the potential for community-based animal sheltering, it is important to examine conditions in the communities currently served by “traditional” animal shelters. Again, Ly et al. used the CIMD, this time to predict the risk of British Columbia residents surrendering their pets to local shelters. The authors found some similarities across parts of the city (e.g., “Situational Vulnerability predicting increased odds of surrendering pit bull-labeled dogs vs. all other dog breeds”) and some differences, “suggesting that provision of animal services, such as veterinary care, for vulnerable groups may be specific to location.”

Using adoption, owner-surrender, volunteer, foster caregiver, and public veterinary service client data from a four-year period (2015–2019), Roberts et al. performed a hot spot analysis across neighborhoods served by the Toronto Humane Society (THS). The authors found that some parts of the city were better served than others, specifically that residents located farther from THS were less likely to utilize the organization's services. Their results provide a framework for developing “strategies to reach under-served demographics.”

According to a 2021 report from the Pet-Inclusive Housing Initiative, 72% of US residents consider pet-friendly housing “hard to find” (7). Combining rental property listings for the 20 most populous cities in Texas with corresponding census tract data, Applebaum et al. examined the issue in greater detail. Their results show that less expensive pet-friendly properties were more likely to charge additional pet fees than were properties that charged higher rents. Moreover, “low-income communities and communities of color were more likely than higher income and predominantly White communities to pay disproportionately higher fees to keep pets in their homes.”

Hoffman et al. report on the results of a May 2021 survey of US households regarding pet ownership and acquisition during the first 14 months of the COVID-19 pandemic. Despite media accounts reporting a dramatic increase in pet acquisition (8, 9), the authors found no significant increase in pet ownership over the study period. In addition, the authors found that pets being rehomed during this period were typically “placed with friends, family members, and neighbors more frequently than they were relinquished to animal shelters and rescues.”

Cárdenas et al. compared two methods for surveying free-roaming dog populations across eight urban and eight rural parishes in Quito, Ecuador: capture-recapture surveys and distance sampling surveys. Each had its limitations—difficulty in identifying individual dogs from photographs, for example, in the case of capture-recapture surveys, difficulties in estimating “animal-observer distances and angles” in the case of distance sampling surveys. As a result, the authors recommend that future studies be conducted *via* “direct observations of dog abundance (number of free-roaming dogs/km) during street counts, complemented with capture-recapture surveys every 5 years.”

To better understand real-world management of free-roaming cats, Aeluro et al. surveyed 567 “feral cat care and advocacy organizations” from across the US. Their findings suggest that many of these organizations have adopted very similar policies and practices (e.g., a minimum weight of 2.0 lbs. for sterilization, left-side ear-tips to indicate sterilization, and less than one quarter engaging in routine feline immunodeficiency virus and feline leukemia virus testing). However, the authors also noted that most of the organizations surveyed lacked clearly defined goals and measures of success.

## Conditions at the intersection of shelter and communityc

As some of the articles included in this Research Topic have highlighted, not all communities served by an animal shelter receive the same level of service (e.g., Ly et al., Roberts et al.). Jenkins and Rudd explore this more deeply, offering a way forward that might be informed by the disability, environmental, gender and sexual diversity, and racial justice movements, among others. The authors finish with a clear call to action: “animal welfare must build authentic relationships with intersectional [Black, Indigenous, and other people of color] communities to holistically address the challenges that impact these communities and their pets. In essence, this work requires the disruption of the status quo within animal welfare to benefit pets within marginalized communities.”

## Conclusions

The original intention of this Research Topic was “to assemble evidence for or against critical concepts, programs, and methods related to community-based animal sheltering and support services” in the hopes that such evidence might “shape the future of animal services.” Specifically, we had in mind a future in which “the interconnectedness of human, animal, and environmental health and welfare outcomes” better inform animal services so that these agencies can “serve their communities in a fair, just, inclusive, and equitable manner.” Again, we have not been disappointed. Indeed, the articles published here represent a notable contribution to the animal welfare literature and—perhaps more importantly—help point the way forward for community-based animal sheltering and support services.

## Author contributions

PW drafted the editorial, which was reviewed, revised, and accepted by all co-authors. All authors contributed to the article and approved the submitted version.

## Conflict of interest

The authors declare that the research was conducted in the absence of any commercial or financial relationships that could be construed as a potential conflict of interest.

## Publisher's note

All claims expressed in this article are solely those of the authors and do not necessarily represent those of their affiliated organizations, or those of the publisher, the editors and the reviewers. Any product that may be evaluated in this article, or claim that may be made by its manufacturer, is not guaranteed or endorsed by the publisher.
